# 

**Published:** 1908-09-19

**Authors:** 


### Forthcoming Books.

Messrs J. and A. Churchill will shortly publish the following new books: "The Cell as the Unit of Life," and other lectures delivered at the Royal Institution, London, 1899-1902 by the late Allan Macfadyen, M.D., B.Sc., Fullerian Professor of Physiology, Royal Institution, London; edited by R. Tanner Hewlett, M.D., F.R.C.P., D.P.H., Professor of General Pathology and Bacteriology, King's College, London. "A Dictionary of Medical Treatment," by Arthur Latham, M.D., F.R.C.P., Physician and Lecturer on Medicine, St. George's Hospital; Senior Assistant Physician, Brompton Hospital for Consumption. "First Lines in Dispensing," by E. W. Lucas, F.I.C., F.C.S., late member of the Board of Examiners of the Pharmaceutical Society of Great Britain.

New editions of the following books are also promised by the same firm: "A Manual of Bacteriology, Clinical and Applied," by R. Tanner Hewlett, M.D., F.R.C.P., D.P.H., Professor of General Pathology and Bacteriology in King's College, London, and Examiner in Pathology, University of Aberdeen. "The Practice of Medicine," by F. J. Charteris, M.B., Ch.B., Assistant to Professor of Materia Medica and Therapeutics, Glasgow University. "An Introduction to the Study of Materia Medica," by Henry G. Greenish, F.I.C., F.L.S., Professor of Therapeutics to the Pharmaceutical Society of Great Britain.

The rate of annual subscriptions to The Hospital, either direct from the Office or through agents, is :—

For the United Kingdom ... ... ... 15s. a year

To the Colonies and Abroad ... ... 17s. a year inclusive of postage in each case.

**Works by GEORGE HERSCHELL, M.D.Lond.,** Senior Physician to the Kensington General Hospital.

**THIRD EDITION. Entirely Re-written. Demy 8vo. price 5s. net; by post 5s. 4d.**

**INDIGESTION:**

**The Diagnosis and Treatment of the Functional Derangements of the Stomach.**

In Preparation. Third Edition. With Illustrations.
**CONSTIPATION AND ITS MODERN TREATMENT.**

Just Published. Second Edition. Demy 8vo. With Illustrations, 1s. 6d. net; by post, 1s. 9d.

**THE DIAGNOSIS OF CANCER OF THE STOMACH:**

With special reference to its recognition in an early stage.

**London: H. J. GLAISHER, 57 Wigmore Street, W.**

THE HOSPITAL September 19, 1908.

*Name* ..............................................................

*Address* ..............................................................

..............................................................

This Coupon must accompany manuscript or contributions intended for The Hospital.

